# Anion- or Cation-Exchange Membranes for NaBH_4_/H_2_O_2_ Fuel Cells?

**DOI:** 10.3390/membranes2030478

**Published:** 2012-07-19

**Authors:** Biljana Šljukić, Ana L. Morais, Diogo M. F. Santos, César A. C. Sequeira

**Affiliations:** Materials Electrochemistry Group, Institute of Materials and Surfaces Science and Engineering, TU Lisbon, Av. Rovisco Pais, Lisbon 1049-001, Portugal; Email: biljana.paunkovic@ist.utl.pt (B.Š.); ana.lucia.morais@ist.utl.pt (A.L.M.); cesarsequeira@ist.utl.pt (C.A.C.S.)

**Keywords:** anion-exchange membrane, cation-exchange membrane, direct borohydride fuel cell, cell performance, cell stability

## Abstract

Direct borohydride fuel cells (DBFC), which operate on sodium borohydride (NaBH_4_) as the fuel, and hydrogen peroxide (H_2_O_2_) as the oxidant, are receiving increasing attention. This is due to their promising use as power sources for space and underwater applications, where air is not available and gas storage poses obvious problems. One key factor to improve the performance of DBFCs concerns the type of separator used. Both anion- and cation-exchange membranes may be considered as potential separators for DBFC. In the present paper, the effect of the membrane type on the performance of laboratory NaBH_4_/H_2_O_2_ fuel cells using Pt electrodes is studied at room temperature. Two commercial ion-exchange membranes from Membranes International Inc., an anion-exchange membrane (AMI-7001S) and a cation-exchange membrane (CMI-7000S), are tested as ionic separators for the DBFC. The membranes are compared directly by the observation and analysis of the corresponding DBFC’s performance. Cell polarization, power density, stability, and durability tests are used in the membranes’ evaluation. Energy densities and specific capacities are estimated. Most tests conducted, clearly indicate a superior performance of the cation-exchange membranes over the anion-exchange membrane. The two membranes are also compared with several other previously tested commercial membranes. For long term cell operation, these membranes seem to outperform the stability of the benchmark Nafion membranes but further studies are still required to improve their instantaneous power load.

## 1. Introduction

Direct borohydride fuel cells (DBFC) are a relatively new type of low-temperature fuel cell that operates using sodium borohydride (NaBH_4_) as the fuel and oxygen (O_2_) or hydrogen peroxide (H_2_O_2_) as the oxidant [1,2,3,4]. This group of fuel cells offers several advantages over conventional proton-exchange membrane fuel cells (PEMFCs), including chemical stability and non-combustibility of the fuel used, simple storage and handling, and significantly lower toxicity of product (sodium metaborate, NaBO_2_) and having the capacity to be recycled to generate NaBH_4_ [5,6]. DBFC operating with H_2_O_2_ as the oxidant, known as direct borohydride/peroxide fuel cells (DBPFCs), are currently being developed and add a further benefit by using a liquid oxidant, whose storage and distribution is much less complicated than gas [7,8].

In the DBPFC, the borohydride (BH_4_^−^) anodic oxidation (Equation 1) proceeds to metaborate (BO_2_^−^) and the direct H_2_O_2_ cathodic reduction (Equation 2) proceeds to H_2_O.

BH_4_^−^ + 8 OH^−^ → BO_2_^−^ + 6 H_2_O + 8 e^−^           E^0^ = −1.24 V *vs.* SHE           (1)

H_2_O_2_ + 2 H^+^ + 2 e^−^ → 2 H_2_O           E^0^ = 1.77 V *vs.* SHE            (2)

This leads to the net cell reaction given by Equation 3, with a theoretical cell voltage of 3.01 V at 25 °C [6].

BH_4_^−^ + 4 H_2_O_2_ → BO_2_^−^ + 6 H_2_O           (3)

However, cell voltages higher than 2 V are rarely achieved in practice. DBPFCs’ performance is determined by anode and cathode materials, electrolytes’ composition, as well as by which membrane separator is used [9]. 

Materials tested as anodes in DBFCs are mainly metals such as platinum (Pt) [10], gold (Au) [11,12], palladium (Pd) [13], silver (Ag) [14], nickel (Ni) [15] and zinc (Zn) [16], as well as their alloys such as Pt-Au [17], Pt-Ag [18], Pt-Ru [19], Pt-rare earth intermetallics [20], Au-Co [21] and Os alloys [22]. Pt is also the most commonly used cathode electrocatalyst for DBFCs [7]. Additionally, Pd [23], Pd-Ir [24], Pd-Ag [25], Pd-Pt [26], Pd-Ru [27], CuO/Nafion/Pt [28], Cu [29], Au [30] and Prussian blue modified electrodes [31] have been studied as cathode materials for H_2_O_2_ electroreduction.

The membrane separator is a crucial component of the fuel cell and plays a double role: it prevents both shorting between anode and cathode and intermixing of anolyte and catholyte. The choice of membrane, its stability and conductivity, determines the electrochemical processes in the cell and the cell’s overall performance [32,33,34,35]. It is known that some cells do not work at all or operate with a much lower efficiency without a separator, as in the case of the Fe/Cr redox and chloralkali cells. When it comes to DBFCs, most DBFCs generally require the use of a membrane separator. Though a membraneless DBFC operating with O_2_ as the oxidant has been recently reported [36], the use of membranes is necessary in DBPFCs since BH_4_^-^ fuel and H_2_O_2_ oxidant immediately react chemically when mixed. 

To attain high cell efficiency, the membrane must satisfy several criteria: high ionic conductivity to provide high currents with minimal resistive losses and minimal or no electronic conductivity, good mechanical strength and stability, chemical and electrochemical stability under operating conditions, adequate moisture, extremely low fuel or oxidant permeability to maximize coulombic efficiency, and cost-effectiveness [37,38]. Still, it is necessary to make a compromise of properties to fulfill the requirement for low internal resistance, good separation as well as adequate physical strength. It has been shown that the membrane thickness has a large impact on cell performance with peak power density increasing with the increase of the membrane thickness [34]. Yet, this influence is rather complex, as thicker membranes will have reduced reactants crossover, *i.e.*, lower NaBH_4_ penetrability, but also higher ionic resistance [38,39,40]. 

**Figure 1:**
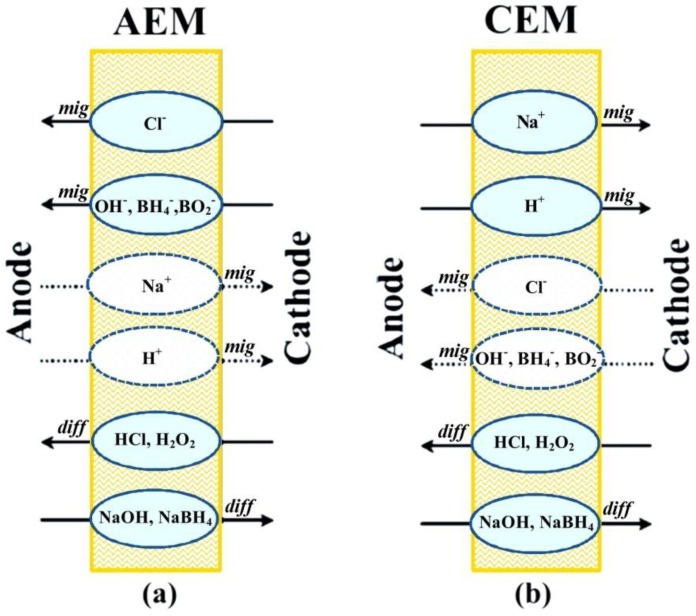
Schematic illustration of the major migrative and diffusive fluxes across (**a**) anion and (**b**) cation exchange membranes used in direct borohydride/peroxide fuel cells (DBPFCs).

Membrane stability under fuel cell operation conditions affects the lifetime and the cost of DBPFCs. In general, both anion-exchange membranes (AEMs) and cation-exchange membranes (CEMs) may be considered as separators for DBPFCs. Membranes operate according to the principle of Donnan exclusion [41], *i.e.*, only transfer of oppositely charged ions is allowed (solid lines in Figure 1), while the transfer of ions of the same charge as the immobilized membrane group is mostly blocked (dotted lines in Figure 1). 

This migrational ion transfer through a membrane is caused by the electric field that builds up as the result of the anodic and cathodic reactions. The charge carrier depends on the type of the used membrane separator. In the case of AEM, OH^−^ and Cl^−^ anions migrate from the cathodic to anodic compartment to keep the charge balance in the cell. Conversely, Na^+^ cations cross through the CEM in the cathode direction maintaining the charge balance. Diffusional crossover of neutral species like NaOH, NaBH_4_, HCl, and H_2_O_2_, gases or organic compounds, due to the concentration gradient between the anode and the cathode compartments, also takes place to a certain extent. For DBPFCs, it has been pointed out that CEMs could be more effective than AEMs in the suppression of BH_4_^−^ crossover due to the ion’s negative charge. Yet, use of CEMs in the DBPFC can lead to a decrease of alkali concentration in the anolyte, causing instability and inefficient use of the NaBH_4_ fuel.

Fuel cell membranes could be divided into four broad categories: perfluorinated ionomers, non-fluorinated hydrocarbons, sulfonated polyarylenes and acid-base complexes [38]. Nafion membranes, from the perfluorinated group, are the major kind of CEMs employed in most of PEMFCs [42], direct methanol fuel cells (DMFCs) [43], as well as in DBFCs [2]. Nafion material meets the requirements of fuel cell membrane such as good ionic conductivity, improved chemical and thermal stability, as well as low fuel permeability [44]. For example, Nafion 117 membrane’s permeability of BH_4_^−^ has been reported to be as low as 8.8 × 10^−9^ mol·cm^−1^·s^−1^ [45]. Consequently, DBFCs using Nafion membranes can achieve peak power densities up to 290 mW·cm^−2^ at 60 °C [46]. Still, Nafion material has some limitations including high cost that impedes the commercialization of fuel cells employing this type of membrane. Thereafter, its replacement by polymer materials with moderate production cost is highly desirable [47,48]. Membranes from the acid-base complexes group are a promising alternative as they have been shown to be competitive to Nafion ones in terms of simplicity and cost of preparation.

In this study, cation- and anion-exchange membranes will be tested as separators in DBPFCs. The performance of laboratory Pt, NaBH_4_/commercial membrane separator/H_2_O_2_ and Pt fuel cells will be evaluated by recording cell polarization, power density and stability curves. These data will be used to calculate important cell parameters, including energy density and specific capacity. Even for commercial membranes, data on their transport properties and performance in fuel cells are limited. The data acquired could be used to improve the design of the membrane separators, *i.e.*, to adjust their properties to yield better cell performance and cell life.

## 2. Experimental Section

All electrochemical measurements were performed using a PAR 273A potentiostat/galvanostat with the PowerSuite software package. A simple two-compartment acrylic cell was used, with each compartment having a volume of 75 cm^3^. Pt electrodes of 1 cm^2^ active area (Metrohm 6.0305.100) served as both anode and cathode. Saturated calomel reference electrodes (SCE, Metrohm 6.0701.100) were employed for evaluation of the anode and cathode overpotentials related to the cell discharge. All experiments were performed at the temperature of 20 ± 2 °C.

Anolyte solution used was 1 M NaBH_4_ (98 wt %, Merck, Darmstadt, Germany) + 4 M NaOH (sodium hydroxide, 99 wt %, Merck, Darmstadt, Germany), while catholyte solution consisted of 3 M H_2_O_2_ (35 wt %, Merck, Darmstadt, Germany) + 1 M HCl (hydrochloric acid, 37 wt %, Panreac, Barcelona, Spain). All chemicals used in this study were of analytical grade and used as received, without further purification. In each experiment, fresh electrolyte solutions were used in order to avoid loss of BH_4_^−^ due to its hydrolysis during solution storage and of H_2_O_2_ due to its decomposition. All solutions were made using deionized water (Elix 3 Millipore). 

Membrane separators investigated were obtained from Membranes International Inc. (Ringwood, NJ, USA) and included one anion-exchange (AMI-7001S) and one cation-exchange (CMI-7000S) membrane. Field emission gun scanning electron microscope (FEG-SEM) JEOL JSM 7001F was used to examine the morphology of both separators. Prior to the cell measurements the membranes were pre-treated by dipping into deionized water for 24 h with water being changed twice during that period, followed by immersion in 4 M NaOH for 2 h. Subsequently, the cation-exchange or the anion-exchange membrane was placed between the acidic and alkaline chambers, with membrane’s active area of *ca.* 30 cm^2^. 

Evaluation of both anion- and cation-exchange membrane for DBPFCs was done by recording polarization as well as power density curves under the same conditions for both separators. Key parameters, including maximum cell voltage, peak power density and short-circuit current density, were assessed. Cell stability tests were performed for DBPFCs employing either membrane, under different operational conditions, allowing the determination of energy densities and specific capacities. First test was done employing constant potential of 0.6 V, while a constant current of 50 mA·cm^−2^ was applied during the second test. Cell durability tests in duration of *ca.* 90 h with no current flow were also performed. Finally, DBPFC performance at a typical current density of 30 mA·cm^−2^ was evaluated until complete cell discharge. 

The characteristics of the studied membrane separators were compared to several other membranes previously tested in our group under the same conditions. These included IONAC MC-3470 and IONAC MA-3475, manufactured by Sybron Chemicals Inc. (Birmingham, NJ, USA), and Nafion N117, Nafion NRE-212 and Nafion 115CS from DuPont (Wilmington, DE, USA).

## 3. Results and Discussion

### 3.1. Membranes Characterization

**Figure 2:**
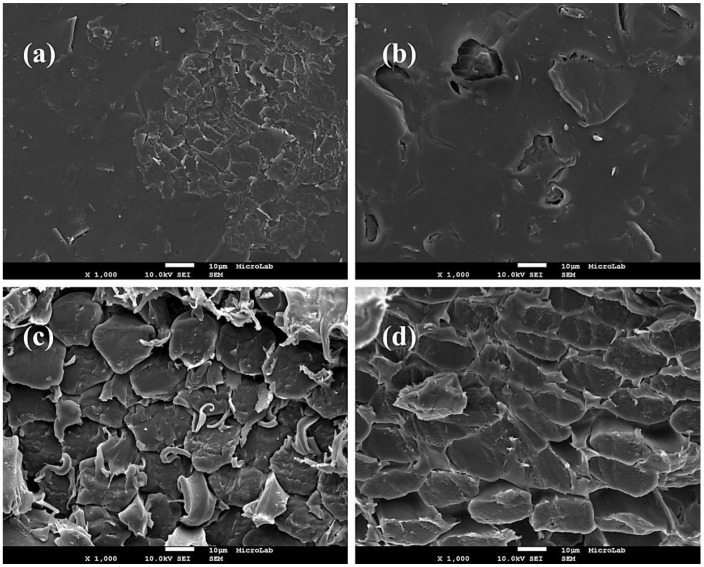
SEM micrographs (×1000) of (**a**) AMI-7001S surface; (**b**) CMI-7000S surface; (**c**)AMI-7001S cross section and (**d**) CMI-7000S cross section.

The morphology of surface and cross section of both anion-exchange membrane AMI-7001S, and cation-exchange membrane CMI-7000S was studied using a FEG-SEM. Figure 2a,b presents the SEM images of the surface of AMI-7001S and CMI-7000S membrane, respectively. These micrographs reveal certain degree of roughness of the surface of both membranes but the topography appears clean of foreign material. 

Figure 2c,d shows the SEM micrographs of the cross sections of AMI-7001S and CMI-7000S membranes, respectively. Presence of densely-packed microfiber in the membranes structure can be observed and, in the case of the anion-exchange membrane, presence of filaments among the microfibers.

Main properties of the studied membranes, anion-exchange AMI-7001S and cation-exchange CMI-7000S membrane, are summarized in Table 1. Both AMI-7001S and CMI-7000S are heterogeneous membranes, based on polystyrene gel cross linked with divinylbenzene, but with different functional groups, *i.e.*, quaternary ammonium and sulfonic acid, respectively. As previously mentioned, membranes are charge selective, AMI-7001S preferably allows migration of anions and CMI-7000S preferably allows migration of cations including H^+^. Limited migration of cations (AEM) and anions (CEM) into the opposite direction can take place as well. 

In terms of properties such as electrical resistance, permselectivity, high total exchange capacity, water permeability, good mechanical and thermal stability, the two studied membranes are similar. Another relevant property of membranes when scaling up fuel cells is their cost. Separators can improve performance, but they also increase total fuel cell price. From cost perspective, there is no difference between the two studied membrane separators. However, it should be noted that the costs for the AMI-7001S and CMI-7000S (€75 per m^2^) membranes are significantly lower than that for the Nafion membranes analyzed in our previous study (Nafion N117, 115CS and NRE-212 with prices of €530, €1025 and €510 per m^2^, respectively) [32].

**Table 1:** Relevant properties of the two studied membrane separators.

	AMI-7001S	CMI-7000S
Membrane type	heterogeneous strong base anion exchange membrane	heterogeneous strong acid cation exchange membrane
Polymer structure	polystyrene gel cross linked with divinylbenzene	
Functional group	quaternary ammonium	sulfonic acid
Ionic form (as shipped)	Cl^−^	Na^+^
Standard thickness (mm)	0.45	
Electrical resistance (Ω cm^2^) 0.5 M NaCl	<40	<30
Permselectivity (0.1 mol KCl kg^−1^/0.5 mol KCl kg^−1^)	90	94
Total exchange capacity (meq·g^−1^)	1.3	1.6
Water permeability (cm^3^·h^−1^·m^−2^ @ 35 kPa)	<32	
Mullen burst strength test (MPa)	>0.62	
Thermal stability (°C)	90	
Membrane cost (€ per m^2^)	75	

### 3.2. Cell Performance Study

**Figure 3:**
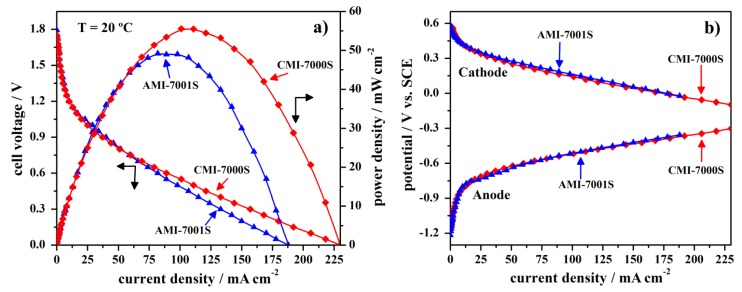
Polarization curves for DBPFCs employing the two tested membranes, including (**a**) cell voltage and power density curves and (**b**) the corresponding electrodes’ potentials.

In order to evaluate the fuel cell performance of the two studied membrane separators, anion-exchange AMI-7001S and cation-exchange CMI-7000S membranes were used to prepare DBPFC. The electrochemical performance of Pt, NaBH_4_/commercial membrane separator/H_2_O_2_, Pt fuel cell was studied using either anion-exchange AMI-7001S membrane or cation-exchange CMI-7000S membrane, with the cell operating at the temperature of 20 °C. The obtained data, *i.e.*, the cell potential *versus* current density curves for DBPFC operating with either AMI-7001S or CMI-7000S membrane separator are shown in Figure 3 and the main obtained parameters are summarized in Table 2.

**Table 2:** Electrochemical parameters of the DBPFCs employing the studied membrane separators.

	Open-circuit voltage (V)	Short circuit current density (mA·cm^−2^)	Peak power density (mW·cm^−2^)	Cell voltage at peak power density (V)	Current density at peak power density (mA·cm^−2^)
AMI-7001S	1.79	188	49	0.60	82
CMI-7000S	1.75	230	56	0.55	101

The observed open circuit voltage (OCV) of both studied DBPFCs is close to 1.8 V, as seen from Figure 3. Peak power density for DBPFC employing CMI-7000S membrane was evaluated to be 56 mW·cm^−2^ at the current density of 101 mA·cm^−2^ at the temperature of 20 °C. Under the same experimental conditions, the DBPFC using AMI-7000S membrane reached slightly lower peak power density of 49 mW·cm^−2^ at the current density 82 mA·cm^−2^. The difference in the performances of DBPFC using CMI-7001S and DBPFC with AMI-7001S membrane was more pronounced in terms of higher short circuit current density attained in the case of CEM-based cell as could be seen from Table 2. These power data indicated CMI-7000S membrane as better separator to be used in DBPFCs.

Recorded electrode potentials for the two DBPFCs do not show a very different behavior. Therefore, the better power performance of the DBPFC employing CMI-7001S membrane separator in comparison with that of DBPFC with AMI-7001S could be attributed to the higher ohmic resistance of the AEM.

The power performance of DBPFCs employing AMI-7001S or CMI-7000S membranes was further compared to the power performance of DBPFCs with previously tested Nafion (N117, 115CS, NRE-212) and IONAC (MC-3470, MA-3475) membranes [32]. The peak power densities of these cells were reported to be between 67 and 96 mW·cm^−2^ at 25 °C, *i.e.*, they were higher than those obtained with the AMI-7001S or CMI-7000S membranes. The DBPFC using a Nafion N117 membrane yielded the best power performance among all the DBPFCs investigated within the two studies. This higher power suggests that the Nafion N117 membrane has a lower ohmic resistance than AMI-7001S and CMI-7000S, possibly due to its smaller thickness (0.18 mm compared to 0.45 mm in case of AMI-7001S and CMI-7000S). Ionic conduction of the membrane depends on its thickness; generally a thinner membrane has a shorter pathway for ion transport and thus a lower ohmic loss.

**Figure 4:**
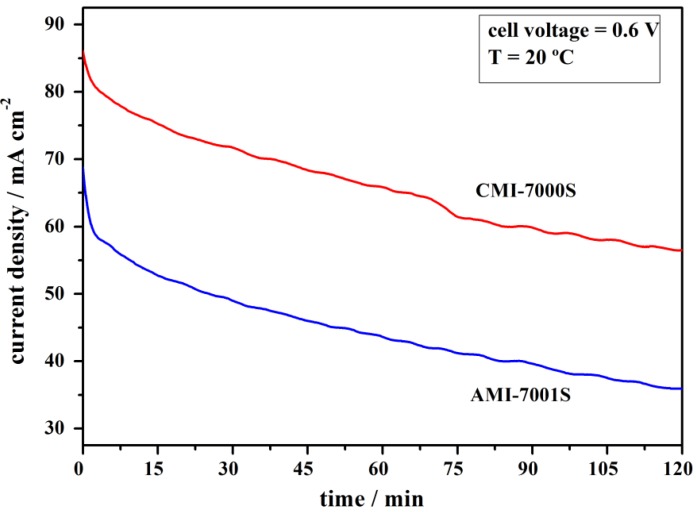
Current density *versus* time at an imposed cell voltage of 0.6 V for DBPFCs employing AMI-7001S and CMI-7000S membrane separators.

Performance of DBPFCs employing each of the two studied membranes was further characterized by imposing a cell voltage of 0.6 V for a period of 2 h (Figure 4). During this period, the measured anode and cathode potentials sustained relatively constant values of about −0.51 V and 0.23 V respectively, for both DBPFCs. Figure 4 shows a slow decay of current density with time in case of DBPFCs employing either of the two studied membranes. However, CMI-7000S membrane–based DBPFC can sustain higher current densities than AMI-7001S–based DBPFC. In comparison with previously studied DBPFCs using different Nafion membranes [32], DBPFCs with AMI-7001S and CMI-7000S can sustain lower current densities, probably due to their much higher thickness. When compared to IONAC membranes, CMI-7000S sustains higher current densities, while AMI-7000S exhibits similar performances as the previously tested IONAC separators.

### 3.3. Cell Stability Study

**Figure 5:**
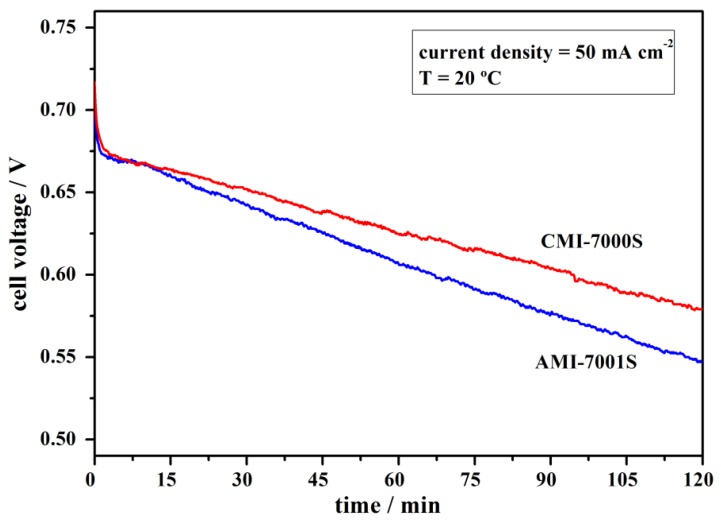
Performance stability of DBPFCs employing AMI-7001S and CMI-7000S membrane separators recorded at the operating current density of 50 mA·cm^−2^.

Performance stability of the Pt, NaBH_4_/commercial membrane separator/H_2_O_2_, Pt fuel cell using AMI-7001S or, alternatively, CMI-7000S membrane was tested both by applying constant current densities and in the absence of current flow. For the constant current measurements, the current density was kept at 50 mA·cm^−2^ for 2 h. As shown in Figure 5, the DBPFCs exhibited a stable performance over the tested period. The operating potential of the cation-exchange membrane DBPFC decreased from the initial potential of 0.70 V to a value of 0.57 V over 2 h. For the anion-exchange membrane based DBPFC the voltage decrease was slightly bigger, *i.e.*, from 0.72 V to 0.55 V over the studied period.

The observed continuous falling of the DBPFCs voltage during cell operation is due to the overpotentials generated inside the cell. These include activation losses due to slow kinetics of the electrochemical reactions at the electrodes, ohmic losses due to resistance of the membrane separator, cell components and interconnects, mass transport losses due to insufficient concentrations of reactants at the electrode/electrolyte interface at high load current condition, and crossover losses due to the crossover of fuel (NaBH_4_) and oxidant (H_2_O_2_) through the membrane. 

**Figure 6:**
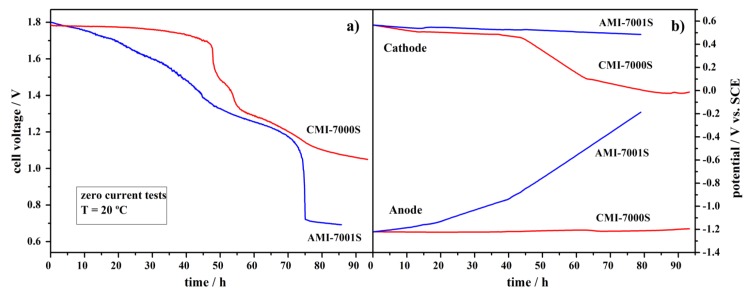
Durability tests for DBPFCs employing AMI-7001S and CMI-7000S membrane separators, at zero current flow, with (**a**) the cell voltage decay and (**b**) the corresponding electrodes’ potentials.

The long-term durability of Pt, NaBH_4_/commercial membrane separator/H_2_O_2_, Pt fuel cells using AMI-7001S or, alternatively, CMI-7000S membrane separators was studied continuously for *ca.* 90 h at no current flow and at the temperature of 20 °C (Figure 6).

As shown in Figure 6a, the CMI-7000S-based DBPFC voltage had constant value of *ca.* 1.77 V for the first 50-h period with the value reducing to 1.05 V during the next 40 h tested. The observed cell voltage decrease followed the same trend as the measured cathode potential values. This decrease of the potential is partially due to the increase of the pH value of the catholyte from *ca.* 0.13 to 4.80. The increase of catholyte pH value is a consequence of several processes occurring in the cell: (i) additional water dragged by Na^+^ ions during their migrational crossover to catholyte compartment; (ii) relatively facile H^+^ diffusional crossover to anolyte compartment; and, probably the most important, (iii) BH_4_^−^ and OH^−^ diffusional crossover to catholyte compartment. The pH change leads to an alteration in the electrode reactions, as H_2_O_2_ reduction mechanism is pH dependent [32], and subsequently leads to a cell voltage decrease. In catholyte solutions of low pH values (pH < 1), the H_2_O_2_ direct electroreduction proceeds through the reaction described by Equation 2.

However, in catholyte solutions of higher pH, H_2_O_2 _is electrochemically reduced via Equation 4, with a much lower standard electrode potential.

H_2_O_2_ + 2 e^−^ → 2 OH^−^ E^0^ = 0.87 V *vs.* SHE           (4)

The produced OH^−^ ions will further increase the pH value of the catholyte solution favoring the H_2_O_2_ reduction described by Equation 3. Also, H_2_O_2_ decomposition (to H_2_O and O_2_) is more pronounced in solutions of higher pH.

AMI-7001S-based DBPFC exhibited worse performance as voltage decrease started immediately from the beginning of the test, leading to a final cell voltage value of 0.71 V in the end of the *ca.* 90 h time period. In this case, the cell voltage showed the same trend as the change of the anode potential value. This large increase in the anode potential values, from −1.2 V to −0.2 V *vs*. SCE (Figure 6b), can be attributed to a significant loss of BH_4_^−^ by diffusional crossover through the AEM, which suffers hydrolysis as soon as it crosses the membrane. It has been reported that the open circuit potential of Pt electrodes is highly dependent on the BH_4_^−^ concentration, allowing their use in sensors for BH_4_^−^ monitoring [49]. The anode potential of −0.2 V *vs*. SCE indicates almost complete BH_4_^−^ depletion. 

As for the catholyte pH change, it was significantly lower than in the case of CEM-based DBPFC, *i.e.*, it increased from 0.13 to only 0.80, as could be expected based on higher electrostatic barrier for H^+^ diffusional crossover imposed by positively charged group of the AEM. It should also be stated that for both CEM and AEM-based DBPFCs, changes on the anolyte pH values were negligible.

The voltage decrease for DBPFCs with AMI-7001S and CMI-7000S was less pronounced than in the case of DBPFCs with the studied Nafion membranes, where cell voltages decrease to a value of about 1.0 V within the first few hours of the test. The slower and less pronounced cell voltage decrease in the case of DBPFCs with AMI-7001S and CMI-7000S membranes is, again, most likely due to the higher thickness of these two membranes when compared to the Nafion ones. Comparing DBPFCs studied herein with the previously explored IONAC membranes, AMI-7001S and CMI-7000S exhibited performance similar to IONAC MC-3470 and somewhat worse than IONAC MC-3475, which sustained a relatively constant cell voltage of 1.6 V during a 50 h test period [32].

**Figure 7:**
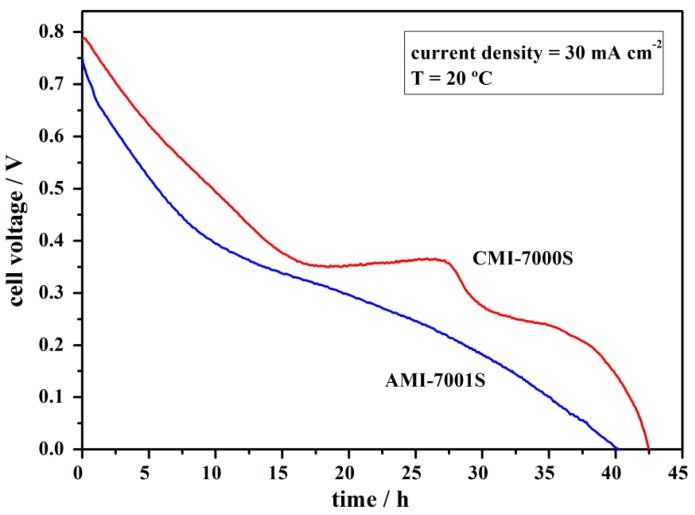
Full discharge of DBPFCs employing AMI-7001S and CMI-7000S membrane separators recorded at the operating current density of 30 mA·cm^−2^.

To simulate practical long-term operation conditions, a constant current density of 30 mA·cm^−2^ was applied to the DBPFCs using each of the tested membrane separators. Cell operation was maintained until achieving complete cell discharge, as can be seen in Figure 7.

At the applied load current density of 30 mA·cm^−2^, DBPFC with CMI-7000S separator sustained higher voltage values than DBPFC with AMI-7001S separator with the observed voltage decreasing close to 0 V after 42 and 40 h, respectively. It was noticed that the faster decay in the voltage of the cell using the AEM membrane is a result of a higher decrease in the cathodic potential value. However, for both cells, the decrease in the anode potential was minimal, which contrasts with Figure 6b. Clearly, the presence of an electric field, which pulls BH_4_^−^ anions towards the anode, helps to prevent the loss of BH_4_^−^ by diffusional crossover.

By integrating the area below the discharge curves it is possible to estimate the energy densities and specific capacities [50]. Energy densities of 129 and 171 W·h·kg^−1^ and specific capacities of 423 and 448 A·h·kg^−1^ were obtained for DBPFCs using AMI-7001S and CMI-7000S membranes, respectively. These values are lower than the theoretically calculated ones [51] but it should be noted that the total NaBH_4_ mass of 2.8 g contained in the anodic compartment, and used in calculations, is higher than the NaBH_4_ mass really consumed during the cell operation.

Typically the membranes are sensitive to OH**^−^** radicals that could be generated during H_2_O_2_ catalytic decomposition as radicals limit durability of the membranes. Therefore it is worth mentioning that no physical/mechanical degradation of the two membranes was visible even after extensive use. Moreover, high reproducibility of the results, *i.e.*, unchanged DBFCs performance, suggested also good chemical stability of both membranes.

Tests performed within this study have shown that CMI-7000S membrane-based DBPFC is able to achieve somewhat higher power density than AMI-7001S-based DBPFC as well as better performance stability and durability. Thus, this CEM separator can be used in DBPFCs to increase power, stability and efficiency, and decrease cost at the same time.

Herein, the membrane separators have been compared by directly evaluating the performance of DBPFCs using either the selected CEM or AEM. Further study of borohydride crossover rates in different ion-exchange membranes is planned for future work, using conductivity measurements together with a potentiometric monitoring method for borohydride determination developed by our group [49]. As mentioned in the Introduction, the borohydride permeability in a Nafion 117 membrane is as low as 8.8 × 10^−9^ mol·cm^−1^·s^−1^. Although this parameter is not known for the membranes studied in the present paper it is predicted that the much higher thickness of the CMI-7000S membrane compared to Nafion 117 will also lead to low borohydride crossover. However, for the positively charged AMI-7001S membrane, the barrier for borohydride crossover is expected to be much lower.

## 4. Conclusions

Relevant properties of an anion-exchange membrane, AMI-7001S, and a cation-exchange membrane, CMI-7000S, as separators in DBPFCs have been reported. Peak power density of 56 mW·cm^−2^ has been observed for the DBPFCs with CMI-7000S membrane at 20 °C. Furthermore, cell performance, stability and durability tests indicated that CMI-7000S leads to a DBPFC with better characteristics operating both in the conditions of constant current and with no-current flow. CEM-based DBPFC could sustain higher voltage/current density in comparison with the AEM-based cell. Energy density of 129 and 171 W·h·kg^−1^ and specific capacity of 423 and 448 A·h·kg^−1^ were obtained for DBPFCs using AMI-7001S and CMI-7000S membranes, respectively, confirming better performance of CEM separator. The analyzed properties of AMI-7001S and CMI-7000s are comparable to those of previously studied similar DBPFCs employing different Nafion and IONAC membranes. DBPFCs with AEM and CEM separator studied herein have somewhat lower power but better stability and durability performance with higher energy density and specific capacity values than those with Nafion and IONAC membranes.
